# The relationship between workaholism and perfectionism among trainee doctors

**DOI:** 10.1192/j.eurpsy.2024.1701

**Published:** 2024-08-27

**Authors:** S. Ellouze, N. Boussaid, A. Mellouli, M. Turki, E. Miledi, N. Halouani, J. Aloulou

**Affiliations:** Psychiatry “B”, Hedi Chaker University Hospital, Sfax, Tunisia

## Abstract

**Introduction:**

In the medical field, work addiction is a double-edged phenomenon. It can be regarded as a positive addiction leading to high motivation to work, but it can also have adverse mental, physical, and social consequences.

**Objectives:**

To assess the relationship between work addiction and perfectionism in trainee doctors.

**Methods:**

We conducted a cross-sectional descriptive and analytical study among trainee doctors. We used the “Work Addiction Risk Test” (WART), and “The Big Three perfectionism scale short form”.

**Results:**

A total of 99 doctors were included. The mean age of participants was 27.6 years, with a sex ratio (M/F) of 0.33. The doctors in our study worked 5.39±1.6 hours a day and were on call 3.84±2.87 times a month. Their average number of hours of sleep was less than 7 hours in 43.4 % of participants. The mean score of the WART was 61.2±14.83. Among the trainee doctors surveyed 39% were considered at high risk of workaholism. The mean WART score was significantly higher among female physicians and those who slept less than 7 hours per day on average. In addition, the average score on the WART scale was significantly associated with the number of calls per month. We found a statistically significant association between perfectionism scores and work addiction scores.

**Conclusions:**

Our study showed that work addiction is common among doctors in training and is favored by high levels of perfectionism. It is therefore essential to explore and define preventive measures to help them find a balance allowing them to aim for high standards and be able to progress, without setting unrealistic expectations, which can lead to work addiction.

**Disclosure of Interest:**

None Declared

